# Determination of Collisional Cross Section Using Microscale High‐Field Asymmetric Waveform ion Mobility Spectroscopy–Mass Spectrometry (FAIMS‐MS)

**DOI:** 10.1002/rcm.10010

**Published:** 2025-02-17

**Authors:** Kristina Krasnova, Colin S. Creaser, James C. Reynolds

**Affiliations:** ^1^ Centre for Analytical Science, Department of Chemistry Loughborough University Loughborough UK

**Keywords:** CCS, FAIMS, Mass spectrometry

## Abstract

**Rationale:**

Collisional cross sections (CCS) are an important characteristic of gas‐phase ions that are measured using ion mobility‐mass spectrometry (IMS). Typically, CCS measurements are performed with drift‐tube IMS or travelling‐wave IMS. However. in a high‐field asymmetric waveform ion mobility (FAIMS) device, ion heating effects make CCS determination more challenging. This research explores whether CCS can be predicted with microscale FAIMS by using known CCS standards.

**Methods:**

An Owlstone ultraFAIMS microscale FAIMS spectrometer was coupled to an Orbitrap Exactive mass spectrometer. Two different CCS standard mixtures (tetraalkylammonium halides [TAAHs] and poly‐DL‐alanine oligomers) were used to evaluate the system's potential to determine CCS. Test peptide bradykinin acetate and substance P were used to evaluate CCS determination accuracy for singly and doubly charged peptide species using external calibration with a series of poly‐DL‐alanine peptides for +1, +2 charge states.

**Results:**

Calibrations with excellent correlation coefficients (*R* ^2^ = 0.99) for both TAAHs and poly‐DL‐alanine were obtained. Good accuracy of determination was achieved for bradykinin [M + 2H]^2+^ with a ± 0.5% difference between experimental and published CCS at a dispersion field (DF) strength of 250 Td; the model proved less accurate for bradykinin [M + H]^+^ (±1.4% at 240 Td). The accuracy of determination for the [M + H]^+^ and [M + 2H]^2+^ ions of substance P was within ± 5% and ± 3% at 250 Td, respectively, while at higher DF values, accuracy decreased to approximately 5%.

**Conclusions:**

Distinct relationships were observed between CCS and transmission CF with both calibrants. Optimum accuracy was obtained at DF 240–260 Td. At lower DF, accuracy is reduced by insufficient resolution of analyte ions from solvent cluster adducts, while at higher DF values, poor transmission becomes a factor. Nevertheless, these data suggest microscale FAIMS can conduct CCS measurements with reasonable accuracy when the compound being measured has similar structural features to the CCS standards used.

## 1 Introduction

Collisional cross sections (CCS) are an important characteristic of gas‐phase ions that can be measured using ion mobility‐mass spectrometry [1]. Determining an ion's CCS can enable more accurate identification and can potentially allow for structural isomers to be distinguished. In addition, by comparing experimentally determined CCS with density functional theory (DFT) modelled structures, experimental confirmation of structures and conformers can be performed, which can prove valuable in experiments where 3D structure and conformation of analytes are important [2, 3].

In the literature, the vast majority of CCS measurements have been performed with either linear drift‐tube ion mobility spectrometry, which enables the direct determination of CCS through the Mason–Schamp equation (Equation 1), where *z* is the charge, *μ* is reduced mass, *T* is the temperature and Ω is the collision cross section, or more recently using travelling‐wave ion mobility spectrometry (TWIMS) where cross sections are determined through comparison of ion mobility with that of known CCS standards [4].

(1) $$K=\frac{3\mathrm{ze}}{16N}{\left(\frac{2\pi }{\mu k_{B}T}\right)}^{0.5}\frac{1}{\Omega }$$

High‐field asymmetric waveform ion mobility spectrometry (FAIMS), also known as differential mobility spectrometry (DMS), is an alternative method that separates ions based on differences in their mobility in the high‐ and low‐field components of an oscillating asymmetric waveform. To achieve this, ions travel between two electrodes at atmospheric pressure under the influence of an asymmetric RF waveform, which is used to generate an electric field, which in the microscale FAIMS device used in this work, typically ranges between 200 and 300 Td (Td = Townsend unit of electric field strength Td = *E*/*N* where *E* is the electric field strength given by applied voltage × electrode gap size [70 μm] and *N* is the number density of the gas), which is formed between electrode plates [5]. This amplitude of this waveform is known as the dispersion field (DF). The high‐field voltage is greater than the low‐field voltage; however, the ions are subjected to the low‐field region for a longer period to ensure an ion trajectory is reversed with the same net force (the product of the voltage and ion trajectory is the same for both components) [6].

Varying mobility behaviour in FAIMS may result from a number of different factors including effective ion temperature, dipole alignment and the clustering and declustering of ion‐neutral clusters in the high‐ and low‐field components of the applied electric field [6]. If the mobility of an ion in the low‐field component is different to its mobility in the high‐field component, the ion will develop a net drift towards one of the electrodes where, on collision with the electrode, the ion will be neutralised. To correct for this, a compensation field (CF) is applied to one of the electrodes to correct the trajectory of the ion, allowing it to traverse the electrodes. Depending on the difference between their high‐ and low‐field mobility, each ion will have a specific CF value, which enables transmission; therefore, by sweeping a range of CF values, ions can be separated from each other based on their specific transmission CF. The differential mobility of ions is dependent on a number of characteristics, including mass, charge, functionality and conformation [7, 8]. For example, the larger the mass of an ion, the less distance the high field will move it from the centre of the FAIMS device. A larger ion will also have a larger CCS, so will undergo more collisions with the buffer gas, which will reduce its mobility [9]. The number of charges on an ion also has a significant effect; the more charges on an ion, then the stronger its interaction with the electric field, so with small molecules, multiply charged species are expected to move farther from the centre of the device and will be observed at higher CF values [7, 10]. In addition to CCS and charge state in FAIMS, the functional groups and conformation of an ion will influence how it interacts with the applied electric field and/or clusters with neutral species such as solvent molecules in the device, which will influence its transmission CF [11, 12]. Subtle differences such as the site at which an ion is protonated can change the behaviour of the ion enough to enable resolution in a DMS as shown by Schorr et al. who used SelexIon DMS to resolve protomers of ciproflaxin [13].

FAIMS has been reported to confer advantages over linear drift tube and/or travelling wave ion mobility in terms of resolving species with similar CCS and may offer a more orthogonal separation because FAIMS resolution is not as closely associated with molecular weight as is observed in other ion mobility methods [14, 15]. Despite these potential advantages, FAIMS has seen relatively little application to the determination of CCS as the CCS of an ion in a FAIMS device is a temperature‐dependent parameter, which may be different in the high‐ and low‐field components of the electric field [16, 17, 18]. However, a key development in the use of FAIMS/DMS to determine CCS occurred in 2021 in the Hopkins lab at the University of Waterloo in Canada [8, 19, 20]. This pioneering work demonstrated that by using a machine learning algorithm and a range of different small molecule calibrants with known CCS, that it was possible to determine the CCS of small molecules, metabolites, lipids and other species with accuracy of < ±2.0%, which is in‐line with the accuracy achieved using linear drift tube and travelling wave methods for CCS determination [21]. This was demonstrated using a planar ABSciex Selexion DMS‐MS system, which uses a 1.5‐cm long drift region before detection with a triple quadrupole mass spectrometer.

**FIGURE 1:**
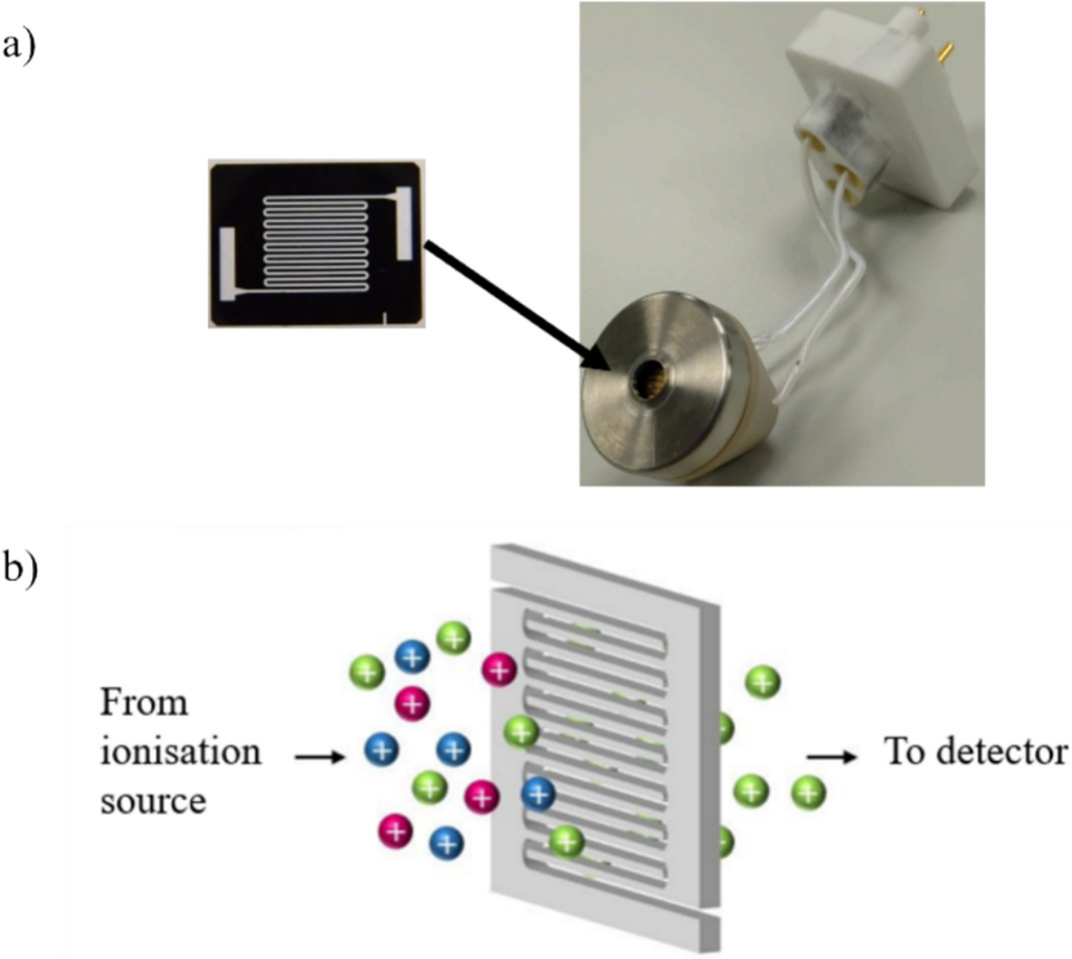
(a) Owlstone miniaturised chip based FAIMS, and (b) schematic diagram of the FAIMS chip showing the ion direction of travel through the device.

The instrument used in this research is a miniaturised high‐field asymmetric waveform ion mobility spectrometer (Owsltone Ultra FAIMS) (Figure 1) [22, 23], which operates at higher electric field strengths and higher frequencies than the DMS system operated by Ieritano et al. The operating parameters of the two devices are vastly different [6, 7, 24, 25]. The microscale FAIMS has a gap size of 70 μm compared with the 0.5 mm gap size in the Selexion; the path length of the device is 700 μm for the Owlstone compared with 1.5 cm for the Selexion device. Therefore, ions will have a much shorter residence time in the device (~100 μs compared with 1.5 ms) [15]. This research aimed to explore whether CCS can be determined using the microscale FAIMS, which will be explored using standards of known collision cross section to determine whether relationships between CCS and transmission CF can be observed.

## 2 Experimental Section

### 2.1 Sample Preparation

Tetraalkylammonium halides (TAAHs), tetrapropyl ammonium chloride, tetrabutyl ammonium bromide, tetrapentyl ammonium bromide, tetrahexylammonium bromide, tetraheptyl ammonium bromide, tetraoctyl ammonium bromide and tetradodecyl ammonium chloride (Sigma‐Aldrich) and a poly‐DL‐alanine (mol wt. 1000–5000) (Sigma‐Aldrich) standard mixture were used to build two CCS calibration models.

Approximately 5 mg of each tetraalkylammonium halide salt was dissolved in 50 mL of 50:50 (v:v) methanol:water and diluted down to a final concentration of 1 μg/mL. For the peptide calibration, 5 mg of poly‐DL‐alanine (Sigma‐Aldrich) was dissolved in 50 mL of 50:50 (v:v) methanol:water +0.1% formic acid before being diluted to a final concentration of 0.1 mg/mL. The tetraalkylammonium halide salts dissociate to form tetraalkylammonium cations (TAA^+^) and halide anions in methanol:water and do not require the typical addition of 0.1% formic acid to promote ionisation during sample introduction to the mass spectrometer. Test peptides (Gly)_5_ bradykinin acetate and substance P (Sigma‐Aldrich) were used to determine the accuracy of determination for the singly charged species and doubly charged species from calibration series of either TAA^+^ ions (Gly_5_) or poly‐DL‐alanine peptides for +1, +2 charge states (bradykinin, Substance P). These peptides were prepared by dissolving approximately 5 mg of test peptide into 50 mL of 50:50 (v:v) acetonitrile:water +0.1% formic acid to produce an 100‐μg/mL solution; this was then diluted down using 50:50 (v:v) acetonitrile:water +0.1% formic acid to a final concentration of 10 μg/mL.

### 2.2 Analysis Method

All analysis was conducted using an Owlstone ultraFAIMS (Owlstone Ltd, Cambridge, UK) coupled to an Orbitrap Exactive (Thermo Fisher). Initial experiments on TAA^+^ ions, poly‐DL‐alanine and the (Gly)_5_ peptide were conducted using electrospray ionisation. Nanoelectrospray ionisation (nano‐ESI) was then explored to reduce the effect of solvent clustering in the FAIMS device. For experiments conducted using the ESI source, calibrants and test peptides were introduced using direct flow injection at 10 μL/min; for nano‐ESI experiments, direct flow injection at 1 μL/min was used. The ultraFAIMS chip was held at 150 °C, and the FAIMS was programmed to cycle a preselected CF range from −3 to 5 Td in a 60‐s sweep. The CF sweep was repeated at DF values starting at 200 Td and finishing at 280 Td stepped up in 10 Td increments. The device is held at atmospheric pressure with measurements taking place in nitrogen.

Ion source conditions (Table 1) were optimised daily for sensitivity; typical source conditions for both ESI and nano‐ESI experiments and the FAIMS conditions used during these experiments are shown in Table 1.

**TABLE 1:** Typical ion source settings and FAIMS parameters used for a direct injection of poly‐DL‐alanine.

Ion source parameters	ESI	Nano‐ESI
Sheath gas flow rate (mL/min)	8	0
Auxiliary gas flow rate (mL/min)	5	0
Sweep gas flow rate (mL/min)	1	2
Spray Voltage (kV)	4.5	2.2
Capillary temperature (°C)	300	300
Capillary voltage (V)	32.5	85
Tube lens voltage (V)	90	150
Skimmer voltage (V)	28	26
FAIMS parameters	Value	
CF start (Td)	−3	
CF end (Td)	5	
CF sweep duration (sec)	60	
DF start (Td)	200	
DF end (Td)	280	
DF step size (Td)	10	

Data processing was performed using the XCalibur software for generation of raw mass spectra; Microsoft Excel was then used for data processing and statistical analysis.

### 2.3 Determination of Transmission CF

**FIGURE 2:**
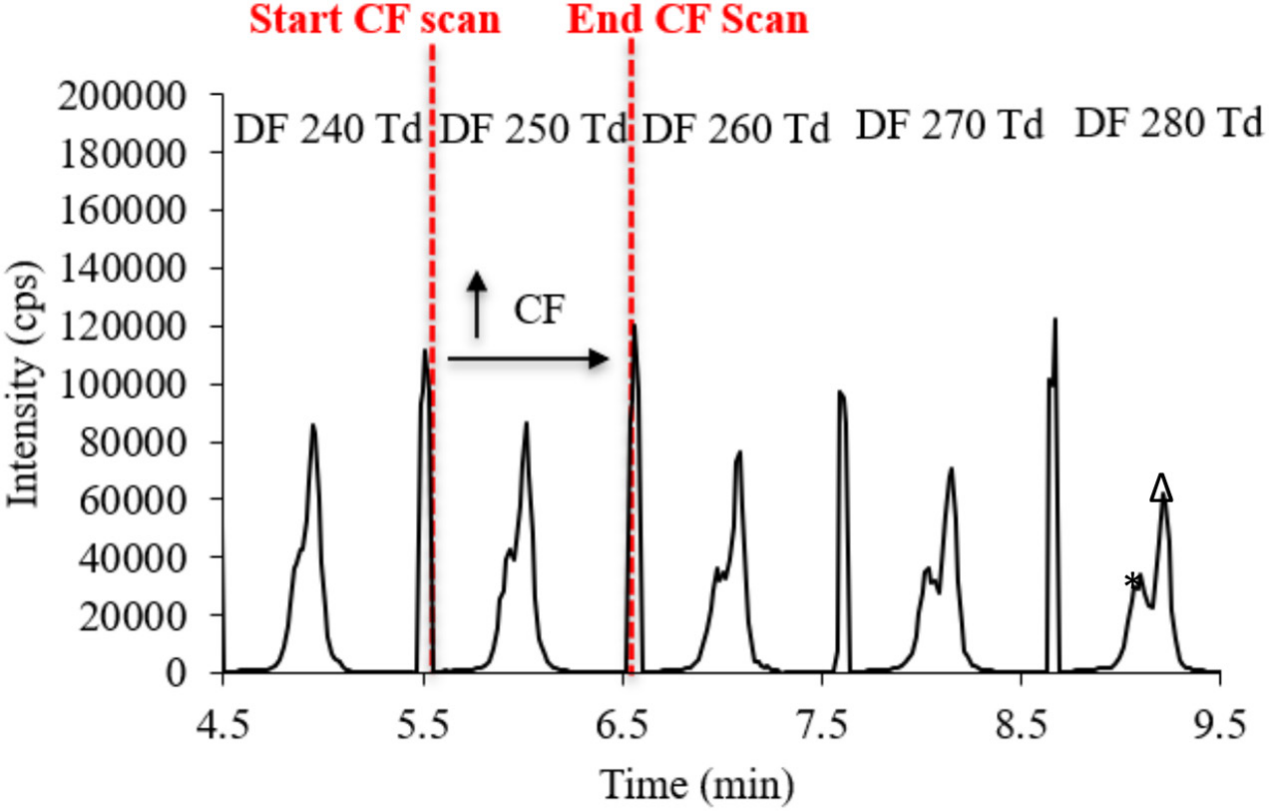
Extracted CF scan for (Alanine)_16_ [M + H]^+^ (*m*/*z* 1155.6122) over the range from −3 to 5 Td at 5 different DF values, where * is [M + H]^+^ solvent clusters and Δ is [M + H]^+^ monomer ions.

To determine the transmission CF for each compound at a specific DF value, a series of calculations were used as the XCalibur software does not have functionality to link the relevant spectra with the DF cycle or CF value that was associated with it. Firstly, the average number of scans the Orbitrap has taken across each DF was found. The total number of scans over the whole DF range is divided by the number of DF values used (Equation 2; Figure 2).

(2) $$\begin{array}{c} \text{Average}\;\mathrm{No}.\text{of scans}\;\mathrm{per}\;\mathrm{DF}=\frac{\text{Total}\;\mathrm{No}.\text{of scans over the full}\;\mathrm{CF}\;\text{sweep}}{\text{number of}\;\mathrm{DF}\;\text{values used}} \end{array}$$

Once the number of MS scans per DF has been calculated, it can be used to determine the CF value that was being used at the time the monomer peak of interest was apparent in the dataset. For an ion at a given DF value, the number of scans in the CF sweep taken to identify the apex of the peak of the monomer can then be divided by the average number of scans per CF sweep calculated above. This is multiplied by the number of CF steps used across the given DF value. To take account of the fact that the CF starting value is in all these experiments is a negative value (either −1 or −3 Td), the starting CF value is then subtracted to give the transmission CF value. (Equation 3; Figure 2).

(3) $$\begin{array}{c} \mathrm{CF}\;\text{value for an}\;\mathrm{ion}\;\mathrm{at}\;a\;\text{given}\;\mathrm{DF}=\\ \left(\left(\frac{\mathrm{No}.\text{of scans to reach the monomer peak apex}}{\text{Average}\;\mathrm{No}.\text{of scans}\;\mathrm{per}\;\mathrm{CF}\;\text{sweep}}\right)\times \mathrm{No}.\text{of}\;\mathrm{CF}\;\text{steps}\;\mathrm{per}\;\mathrm{CF}\;\text{sweep}\right)-\text{starting}\;\mathrm{CF}\;\text{value} \end{array}$$

## 3 Results and Discussion

### 3.1 Tetra‐Alkyl Ammonium Halide (TAAH) Calibrants

All tetraalkylammonium cations (TAA^+^) were successfully detected using the FAIMS‐MS system with electrospray ionisation and can be observed in Figure S1 at *m*/*z* 186.2262 (tetrapropyl), 242.2901 (tetrabutyl), 298.3540 (tetrapentyl), 354.4179 (tetrahexyl), 410.4819 (tetraheptyl) 466.5460 (tetraoctyl) and 690.8016 (tetradodecyl), respectively; published cross sections for these ions were taken from the McLean group; unified CCS compendium are shown in Table S1 [26].

Optimisation focussed on the application of DF strengths between 180 and 300 Td (Townsend) was completed using TAA^+^ ions. However, the anticipated tetradodecylammonium monomer was not readily observed at 180 Td due to lack of separation from solvent cluster ions. The smallest ion (tetrapropylammonium) investigated at the highest DF strength of 300 Td was not observed as this small ion was being displaced by such a significant amount in the high field that its intensity had dropped to the point it could no longer be detected. This is likely due it either being displaced so much that it impinges on the electrodes of the FAIMS system and neutralises or dissociating due to field induced heating effects. While the initial method worked well for a specific range of masses, it would not be suitable for larger molecules such as proteins and lipids; therefore, it was decided to move to a narrower DF range, between 200 and 280 Td, using steps of 10 Td, to generate calibration data.

**FIGURE 3:**
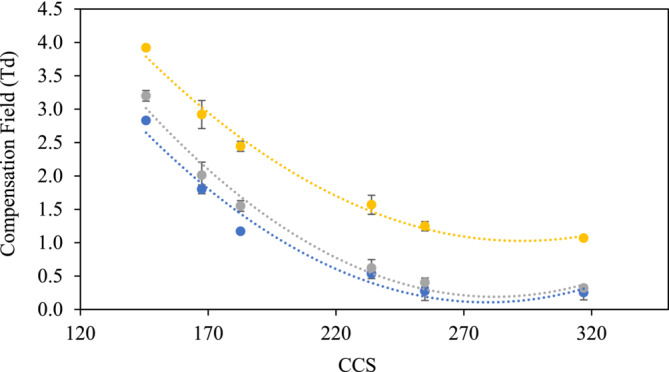
The calibration graph of CF values against CCS from second order polynomial fit model for each of the TAA^+^ cations using a DF value of 200 Td (blue), 230 Td (grey) and 280 Td (yellow).

Calibrations of transmission CF versus CCS for the TAA^+^ ions were obtained at each of the DF values tested, and plots of CF versus CCS were generated (Figure 3). Standard deviation of repeated measurements for each data point was used to generate error bars.

The plots shown in Figure 3 were constructed without using the tetrahexylammonium halide ion (which was treated as an unknown). Polynomial, exponential, power and logarithmic fittings were fitted to calibration data obtained from both TAA^+^ and poly‐DL‐alanine calibrants, and it was found that the polynomial fitting gave the most consistently good correlation coefficients so was used for all data obtained. When applying the polynomial fitting to the electrospray TAA^+^ calibrant data, each of the plots gave good correlation coefficients (*R* ^2^ 0.97 to 0.99). The graphs produced (Figure 3) were then used to determine the cross section of the tetrahexylammonium ion and a (Gly)_5_ test peptide by comparing the experimentally measured value with published values from the unified CCS compendium [26].

The data obtained for the CCS determination of the tetrahexylammonium ion showed that reasonable accuracy was obtained across the range from DF 220 Td to DF 260 Td (Table S2) with an average error of 3.2%. The CCS determination for the (Gly)_5_ ion however consistently overestimated the CCS at all DF values explored (Table S3) with an average error of 22% across the full CF range. This difference is shown visually on Figure S2, which shows a CCS versus transmission CF plot of the calibration obtained at 260 Td with the positions of the tetrahexylammonium ion and the (Gly)_5_ ion indicated. This observation tallies with research conducted on CCS measurements using TWIMS; in both FAIMS CCS determination and TWIMS, the CCS is determined indirectly using a calibration procedure and optimal accuracy is achieved only when the CCS calibration standards closely match the structure of the analyte.

To attempt to improve the accuracy of the CCS determination, a subsequent calibration series was performed using nano‐ESI; the Owlstone ultraFAIMS system used in this research is positioned before the heated inlet capillary of the mass spectrometer, and due to this source geometry, clustering of analyte ions with solvent molecules is frequently observed in FAIMS‐MS data.

The accuracy of the method was improved when using nano‐ESI, and the highest accuracy was achieved using the calibration obtained at 260 Td, which yielded an experimental CCS within ± 0.02% of the published value shown in Table S4. This result is in‐line with the level of accuracy expected from using collision cross‐section determination using the MobCal‐MPI CCS determination method used in drift‐tube and travelling wave ion mobility–mass spectrometry, which shows a predictive accuracy of 2.2% [21]. However, when looking at the accuracy of CCS determination at other DF values, there is variation in the accuracy of the calculated cross section at each different DF value, with the mean accuracy of prediction when considering DF 220–280 Td giving an average accuracy of ± 1.4%, which is matching the accuracy of T‐wave CCS determination, showing the potential for CCS determination of closely related compounds. This agrees with Ieritano's observations and suggests a microscale device can also be used to determine CCS.

The device was then applied to study peptide calibrants to determine whether larger species or multiply charged species can also show a correlation between transmission CF and CCS. Poly‐DL‐alanine was selected as a peptide calibrant as it has been widely reported as a calibrant mixture in T‐wave CCS determination experiments, and because as a relatively nonpolar peptide with a low dipole moment, it should show similar properties to the TAAH calibrants.

### 3.2 Poly‐DL‐Alanine Peptide Calibrant

Poly‐DL‐alanine calibration standards and the test peptides (bradykinin and substance P) were analysed using both ESI and nano‐ESI ionisation; however ESI demonstrated extensive clustering of calibrant and test analyte ions with solvent molecules, as such all subsequent CSS determinations were conducted using nano‐ESI. Calibration plots obtained with nano‐ESI were significantly different from those obtained with ESI (Figure S3) with calibrants of low CCS being consistently transmitted at higher DF values when ionised with nano‐ESI than when ionised with ESI. These data show that the Owlstone device is highly sensitive to the presence of solvent vapours, and when using a microscale device to conduct CCS measurements, the sample flow rate in the device needs to be consistent to avoid introducing additional error into the calibration.

**FIGURE 4:**
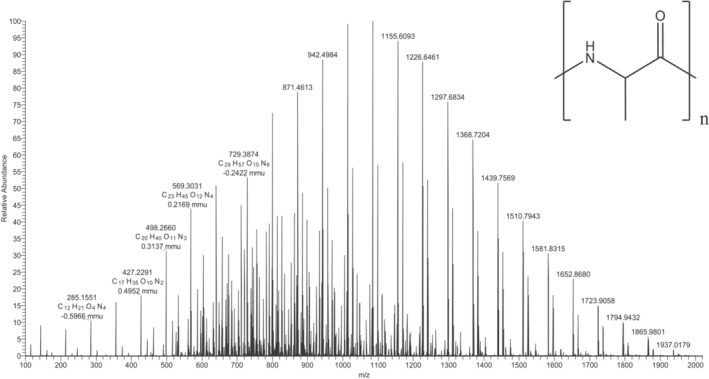
ESI‐MS average mass spectra of poly‐(DL)‐alanine solutions at 100 μg/mL.

The nano‐ESI‐MS analysis of the poly‐DL‐alanine sample produced a complex mass spectrum consisting of a range of singly and multiply charged (2^+^ and 3^+^ charge state ions) and can be seen in Figure 4. The charge state of ions was confirmed using their isotope distributions. Published cross sections taken from the unified CCS compendium [26] for the +1, +2 and +3 charge state poly‐DL‐alanine oligomers are shown in Tables S5, S6 and S7 respectively.

**FIGURE 5:**
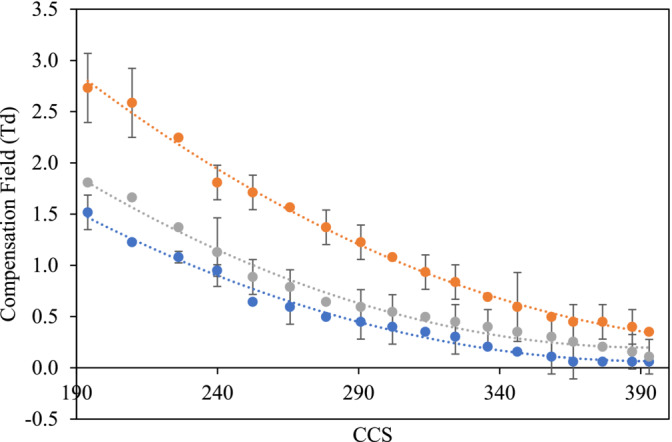
The calibration graph of CF values against CCS from polynomial fit model of singly charged poly‐DL‐alanine cations using a DF value of 200 Td (blue), 220 Td (grey) and 250 Td (orange).

**FIGURE 6:**
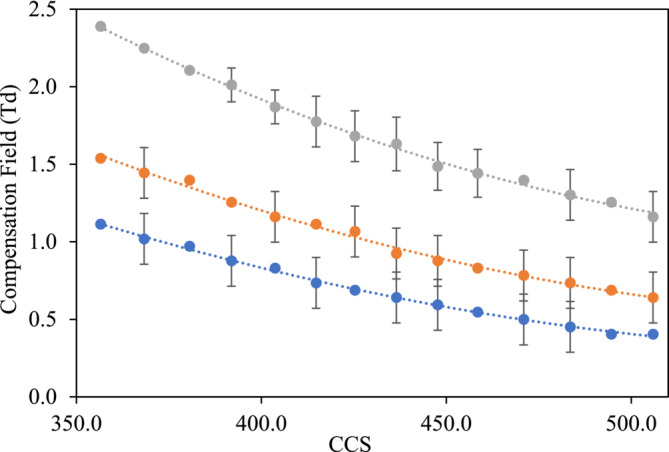
The calibration graphs of CF values against CCS from polynomial fit model of doubly charged poly‐DL‐alanine cations using a DF value of 200 Td (blue), 220 Td (orange), 250 Td (grey).

A calibration series of poly‐DL‐alanine peptides was obtained, which produced calibrations for +1 (Table 2 and Figure 5), +2 (Table S8 and Figure 6) and +3 (Table S9 and Figure S4) charge states. These calibrations also showed clear relationships between CCS and transmission CF for all three charge state series (*R* ^2^ 0.983 to 0.999), covering a range of CCS from (194 to 393 Å^2^, 356.5 to 505.9 Å^2^ and 578.2 to 649.3 Å^2^ for the +1, +2 and +3 charge states, respectively).

**TABLE 2:** The measured CF values for singly charged poly‐DL‐alanine cations at DF value of 250 Td.

Poly‐DL‐Alanine	*CCS* Å^2^	Mean CF (Td)
(Alanine)_6_	194.0	2.7316
(Alanine)_7_	209.7	2.5859
(Alanine)_8_	226.2	2.2459
(Alanine)_9_	239.9	1.8087
(Alanine)_10_	252.5	1.7116
(Alanine)_11_	265.7	1.5659
(Alanine)_12_	278.5	1.3716
(Alanine)_13_	290.8	1.2258
(Alanine)_14_	302.0	1.0801
(Alanine)_15_	313.7	0.9344
(Alanine)_16_	324.3	0.8373
(Alanine)_17_	335.8	0.6916
(Alanine)_18_	346.3	0.5944
(Alanine)_19_	358.4	0.4973
(Alanine)_20_	366.1	0.4487
(Alanine)_21_	376.5	0.4487
(Alanine)_22_	387.0	0.4001
(Alanine)_23_	393.0	0.3515

The calibration shown in Figure 5 shows the transmission CF for each of the poly‐DL‐alanine [M + H]^+^ ions plotted against their published values from the database Unified CCS Compendium by McLean research group [26]. This figure shows the calibrations obtained from singly charged poly‐DL‐alanine ions at three different DF values (200, 220 and 250 Td). It can be clearly seen that as the DF increases, there is a corresponding increase in transmission CF for all of poly‐DL‐alanine [M + H]^+^ ions. Overall, the calibrations showed a clear relationship between CCS and transmission CF for all three charge state series investigated (*R* ^2^ 0.979 to 0.999), covering a range of CCS from (194 to 393 Å^2^, 356.5 to 577.6 Å^2^ and 578.2 to 649.3 Å^2^ for the +1, +2 and +3 charge states, respectively).

To determine the accuracy of the model, test peptides, bradykinin and substance P were used to validate the model by determining their CCS using the calibrations obtained from the poly‐DL‐alanine ions and then comparing their experimentally measured values with published values from the Unified CCS Compendium [26].

The [M + H]^+^ (*m*/*z* 1060.569) and [M + 2H]^2+^ (*m*/*z* 530.7886) ions of bradykinin were used to determine accuracy. The CCS measurements of [M + H]^+^ and [M + 2H]^2+^ ions of bradykinin from the Unified CCS Compendium are 314.6 and 344.2 Å^2^, respectively. The primary structure of bradykinin is a 9‐amino acid peptide chain. The sequence of its amino acids is Arg‐Pro‐Pro‐Gly‐Phe‐Ser‐Pho‐Phe‐Arg [7]. Good accuracy was obtained for the singly charged species using the calibration obtained at 250 Td, which obtained an experimental CCS within ± 1.4% of the published value (Table 3) with accuracy across all DF values ranging between ± 1.4% to ± 5.7%. There are a number of potential causes for inaccuracy in the model; it may arise from poor resolution of the bradykinin [M + H]^+^ ion from cluster ion species; the presence of different conformers of bradykinin, which can overlap; or from the lack of separation between the higher CCS calibrant ions at low DF values, which is evidenced by the shallower gradients observed at 200 and 220 Td when compared with the gradient obtained at 250 Td in Figure 5. With a shallower gradient, the effect of any error will be magnified giving a greater variation in the experimentally determined CCS.

**TABLE 3:** The percentage change in CF of the singly charged bradykinin ion [M + H]^+^ between experimentally calculated CF value and those derived from polynomial fit model using poly‐DL‐alanine.

DF (Td)	Mean CF (Td)	Predicted CCS (Å^2^)	Difference (Å^2^)	% change
200	0.3006	319.0	4.1	1.3954
210	0.4054	303.5	−11.1	−3.5149
220	0.5102	301.9	−12.7	−4.0397
230	0.6149	303.4	−11.2	−3.5731
240	0.7197	319.1	4.5	1.4196
250	0.8245	329.5	14.9	4.7448
260	0.9286	331.1	16.5	5.2467
270	1.0864	321.1	6.5	2.0559
280	1.1388	332.6	18.0	5.7193

Doubly charged calibrant ions showed a marked shift to higher transmission CF when compared with singly charged species. For example, the average transmission CF value of the singly charged (Alanine)_16_ was calculated as 0.8373 Td at DF of 250 Td (Table 3); however, at the same DF value, the average transmission value of the doubly charged (Alanine)_16_ was determined to be 2.3901 Td (Table S8). The effect of the electric field on an ion is influenced by the number of charges on an ion, so increasing the number of charges will increase the acceleration of the ion in the high and low field components of the asymmetric waveform. Therefore, doubly charged ions will experience a higher degree of field induced heating than its singly charged analogue, which will induce structural changes (e.g., unfolding) at high field. These effects will impact its differential mobility to a higher degree than the high‐field impact on a singly charged species resulting in a shift to higher transmission CF [7].

The [M + H]^+^ and [M + 2H]^2+^ ions of bradykinin were then used to determine the accuracy of experimentally determined CCS; good accuracy was obtained for the doubly charged species with a ± 0.5% difference between experimental and published CCS at 250 Td, which was better than the accuracy achieved for the singly charged species (± 1.4% at 240 Td) (Tables 3 and 4).

**TABLE 4:** The percentage change in CF of the doubly charged bradykinin ion [M + 2H]^2+^ between experimentally calculated CF value and those derived from polynomial fit model using poly‐DL‐alanine.

DF (Td)	Mean CF (Td)	Predicted CCS (Å^2^)	Difference (Å^2^)	% change
200	1.4000	324.7	19.5	5.6769
210	1.6101	300.4	43.9	12.7199
220	1.8723	322.3	21.9	6.3675
230	2.1866	319.7	24.5	7.1252
240	2.3438	334.2	10.0	2.8913
250	2.5010	342.3	1.9	0.5494
260	2.9725	338.0	6.2	1.7920
270	3.2868	352.3	−8.1	−2.3530
280	3.6012	350.7	−6.5	−1.8911

**FIGURE 7:**
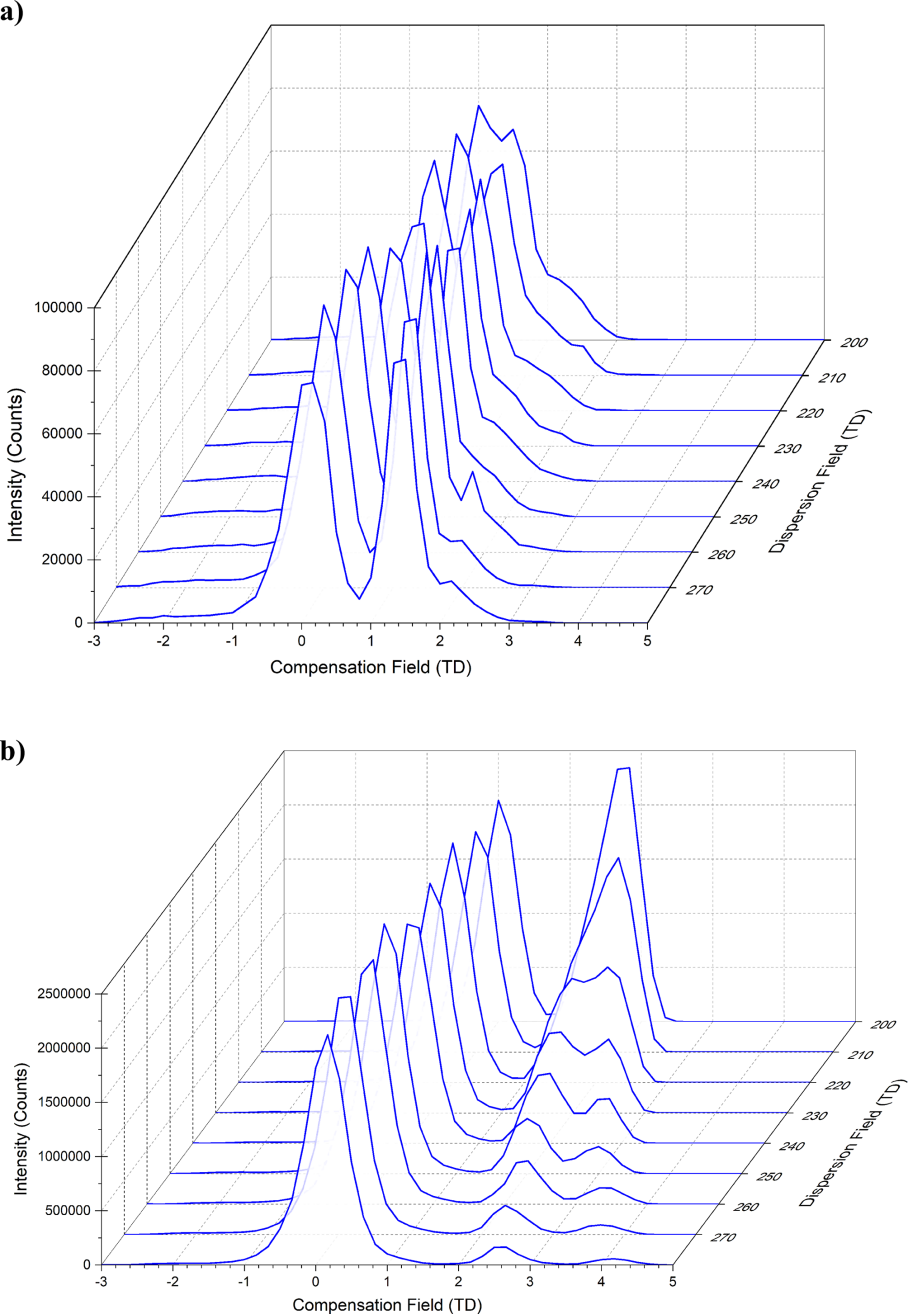
FAIMS‐MS DF versus CF waterfall plot for (a) bradykinin [M + H]^+^ ions and (b) bradykinin [M + 2H]^2+^ ions at DF values ranging from 200 to 280 Td, cycling through a CF range from −3 to 5 Td.

The most likely cause for inaccuracy when determining CCS at lower DF values is poor resolution of the desired ion from cluster ion species. At higher DF values with the bradykinin [M + 2H]^2+^ ions, the presence of different conformers is observed. FAIMS has particular utility for the separation of conformers, which can be useful when studying biomolecules such as proteins [27]. Drift‐tube ion mobility mass spectrometry studies conducted by the Clemmer group showed that bradykinin can adopt up to 10 different gas phase conformers, which are influenced by the composition of the solvent in which the bradykinin is dissolved [28, 29, 30]. The presence of different conformers of bradykinin has also been reported using a microscale FAIMS device [7]. Figure 7 shows waterfall plots of the extracted ion traces for both the singly and doubly charged bradykinin at DF values ranging from DF 200 to 280 Td. The [M + H]^+^ monomers of bradykinin were not clearly resolved at 200–230 Td due to lack of separation from solvent cluster ions (Figure 7). However, the data obtained suggests this does not significantly reduce the accuracy of CCS determination. In contrast, at 200 Td, the CF scan showed two distinct peaks for the [M + 2H]^2+^ monomer ions and solvent cluster ions of bradykinin (Figures 7 and S5). As the DF increases for the singly charged bradykinin, separation from the cluster ions can be observed; however, it is worth noting the shape of the second peak, which appears to have a shoulder on the front edge, which may be indicative of the presence of two different conformers of bradykinin. A similar effect can be observed with the doubly charged bradykinin, while here, this gives a good separation between clustered and un‐clustered bradykinin; two different peaks can be observed to resolve from each other as the DF increases [7]. The measurements presented in Table 4 are calculated using the conformer, which is transmitted at higher CF. At higher DF values, the separation of these two proposed conformers can enable an accurate determination when using the peak at the highest transmission CF. An issue here is observed when operating at high DF values in that ions are lost through diffusion, neutralisation on the electrodes or dissociation due to field induced heating. These effects cause intensity to drop significantly, in some cases, up to the point the ion could no longer be detected [31]. It is important, therefore, that the correct conformer is selected when performing CCS determination. For some species, DFT modelling may be needed to determine which conformer is responsible for a particular peak.

Similar behaviour was observed for [M + H]^+^ and [M + 2H]^2+^ ions of substance P (Tables S10 and S11; Figure S6). The accuracy for singly charged substance P was significantly worse with an accuracy of ± 5% between experimental and published CCS at 250 Td; at lower DF values, accuracy was significantly degraded with accuracy ranging between ± 9.1% and ± 13.6% at DF 200–240 Td. This most likely results from poor resolution of the substance P [M + H]^+^ ion from solvent cluster ions in the device, which can be observed in Figure S4. Similar to the observations with bradykinin ions, the prediction accuracy for the substance P [M + 2H]^2+^ ion is significantly better than that achieved for the singly charged species, a prediction accuracy of ± 1.6% achieved at 240 Td, with prediction accuracy dropping to ± 5% high DF values. Figure S4 shows that the substance P [M + 2H]^2+^ ion is better resolved from solvent cluster ions than the [M + H]^+^ ion, which may be a contributing factor to the improved accuracy for the doubly charged ions.

## 4 Conclusions

Relationships were observed between CCS and transmission CF for tetraalkylammonium halide ions, and both singly and multiply charged peptide ions. Using second‐order polynomial functions, calibrations with excellent correlation coefficients (*R* ^2^ = 0.99) could be obtained. The applied DF value has a distinct effect on how well calibration works over a specific CCS range, with optimum performance over the widest range of CCS values obtained at DF 240–260 Td. Peptides can also form multiple conformers as shown with bradykinin. FAIMS can separate conformers, and whether it can determine the CCS of different conformers accurately would require further investigation. The data presented suggest that microscale FAIMS can conduct CCS measurements with reasonable accuracy and precision when the species being measured has similar structural features, geometry, and functionality to the CCS standards used. However, when there are differences in structure and polarity even amongst ions of the same species such as peptides, the accuracy of the CCS determination is reduced. Poly‐DL‐alanine is a relatively nonpolar molecule with methyl group side chains. They are hydrophobic and will have relatively weak interactions with water and polar solvent molecules. Peptides with different structures will have different interactions with various solvents, which may impact on their separation during FAIMS process. These differences in structure and behaviour will thus influence how the ion behaves in the FAIMS device. This suggests a sequence of non‐polar peptides like poly‐DL‐alanine may not work well when trying to determine the CCS of highly polar peptide species. The data shown suggests that CCS standards should possess similar structural motifs and polarity to the analyte being measured if an accurate determination is to be obtained. For example, if carrying out CCS measurements on organic amines, then organic amine CCS standards should be used if accurate determination is to be achieved [7, 32, 33]. The data presented here shows predictive trends amongst the two classes of compounds investigated and suggests that the fundamental description of differential mobility proposed by Ieritano et al. [20] with the Selexion DMS holds true for the microscale Owlstone device. However, a larger scale study using a diverse range of analytes with different chemistry would be required in order to develop a generalisable model such as that demonstrated by Ieritano et al. using the Selexion [20]. If the mass spectrometer can assign a putative compound class based on accurate mass *m*/*z* or fragmentation information, then the analyte could be compared with the appropriate set of standards for CCS determination. This approach has merit for small molecules, but it is more challenging for peptide analytes as multiple charges, post translation modifications and differences in sequence could all influence the mobility and thus reduce the accuracy of any CCS determinations.

## Author Contributions

**Kristina Krasnova:** methodology, visualization, writing – original draft, formal analysis, data curation. **Colin S. Creaser:** conceptualization, writing – review and editing. **James C. Reynolds:** supervision, conceptualization, writing – review and editing.

### Peer Review

The peer review history for this article is available at https://www.webofscience.com/api/gateway/wos/peer‐review/10.1002/rcm.10010.

## Supporting information


**Figure S1:** ESI‐MS average mass spectra of TAAH solutions at 1 μg/mL.
**Table S1:** Published CCS of tetraalkylammonium compounds (TAAHs) measured in nitrogen gas.^22^
**Table S2:** The percentage change in CCS of the tetrahexylammonium halide ion between the published CCS value and those derived from a second‐order polynomial fit model using TAA^+^ calibrant ions with electrospray ionisation.
**Table S3:** The percentage change in CCS of the (Gly_5_) [M + H]^+^ ion between the published CCS value and those derived from a second‐order polynomial fit model using TAA^+^ calibrant ions with electrospray ionisation.
**Figure S2:** Calibration graph of transmission CF values versus CCS for TAA^+^ calibrant ions obtained at a DF value of 260 Td and used for CCS determination of the C6 TAA^+^ and (Gly)_5_ ions.
**Table S4:** The percentage change in CCS of the tetrahexylammonium halide ion between the published CCS value and those derived from a second‐order polynomial fit model using TAA^+^ calibrant ions with nano‐electrospray ionisation.
**Table S5:** Published CCS of singly charged Poly‐DL‐alanine cations measured in nitrogen gas and their *m*/*z*.^22^
**Figure S3:** Overlaid calibration data from ESI‐FAIMS‐MS and nano‐ESI FAIMS‐MS analysis of poly‐DL‐alanine obtained at a DF value of 280 Td.
**Table S6:** Published CCS of doubly charged poly‐DL‐alanine cations measured in nitrogen gas and their *m*/*z*.^22^
**Table S7:** Published CCS of triply charged poly‐DL‐Alanine cations measured in nitrogen gas and their *m*/*z*.^22^
**Table S8:** Calculated transmission CF values for doubly charged poly‐DL‐alanine cations at DF value of 250 Td.
**Table S9:** Calculated CF values for triply charged poly‐DL‐alanine cations at DF value of 250.
**Figure S4:** Calibration graphs of CF values versus CCS from polynomial fit model of triply charged poly‐DL‐alanine cations using a DF values of 200 Td (blue), 220 Td (orange) and 250 Td (green).
**Figure S5:** Full extracted CF scan for (a) the singly charged Bradykinin ion [M + H]^+^ (blue), where * is [M + H]^+^ solvent clusters and Δ is [M + H]^+^ monomer ions, and (b) the doubly charged Bradykinin ion [M + 2H]^2+^ (orange) over the range from −3 to 5 Td at nine different DF values, where * is [M + 2H]^2+^ solvent clusters and Δ and ○ are [M + 2H]^2+^ monomer ions, which are conformational isomers of each other.
**Table S10:** The percentage change in CCS of the singly charged substance P [M + H]^+^ between the published CCS value and those derived from polynomial fit model using poly‐DL‐alanine.
**Table S11:** The percentage change in CCS of the doubly charged substance P ion [M + 2H]^2+^ between the published CCS value and those derived from polynomial fit model using poly‐DL‐alanine.
**Figure S6:** Full extracted CF scan for (a) the singly charged substance P ion [M + H]^+^ (blue), where * is [M + H]^+^ solvent clusters and Δ is [M + H]^+^ monomer ions, and (b) the doubly charged substance P ion [M + 2H]^2+^ (orange) over the range from −3 to 5 Td at nine different DF values, where * is [M + 2H]^2+^ solvent clusters and Δ is [M + 2H]^2+^ monomer ions.

## Data Availability

The data that supports the findings of this study are available in the supplementary material of this article.
