# Supplementary material for: Determination of Collisional Cross Section Using Microscale High‐Field Asymmetric Waveform ion Mobility Spectroscopy–Mass Spectrometry (FAIMS‐MS)
Source: Rapid Commun Mass Spectrom. 2025 Feb 17;39(10):e10010. doi: 10.1002/rcm.10010 (PMC11832801; doi:10.1002/rcm.10010)
Supplement: Supplementary file 1 — Figure S1: ESI‐MS average mass spectra of TAAH solutions at 1 μg/mL. Table S1: Published CCS of tetraalkylammonium compounds (TAAHs) measured in nitrogen gas.22 Table S2: The percentage change in CCS of the tetrahexylammonium halide ion between the published CCS value and those derived from a second‐order polynomial fit model using TAA+ calibrant ions with electrospray ionisation. Table S3: The percentage change in CCS of the (Gly5) [M + H]+ ion between the published CCS value and those derived from a second‐order polynomial fit model using TAA+ calibrant ions with electrospray ionisation. Figure S2: Calibration graph of transmission CF values versus CCS for TAA+ calibrant ions obtained at a DF value of 260 Td and used for CCS determination of the C6 TAA+ and (Gly)5 ions. Table S4: The percentage change in CCS of the tetrahexylammonium halide ion between the published CCS value and those derived from a second‐order polynomial fit model using TAA+ calibrant ions with nano‐electrospray ionisation. Table S5: Published CCS of singly charged Poly‐DL‐alanine cations measured in nitrogen gas and their m/z.22 Figure S3: Overlaid calibration data from ESI‐FAIMS‐MS and nano‐ESI FAIMS‐MS analysis of poly‐DL‐alanine obtained at a DF value of 280 Td. Table S6: Published CCS of doubly charged poly‐DL‐alanine cations measured in nitrogen gas and their m/z.22 Table S7: Published CCS of triply charged poly‐DL‐Alanine cations measured in nitrogen gas and their m/z.22 Table S8: Calculated transmission CF values for doubly charged poly‐DL‐alanine cations at DF value of 250 Td. Table S9: Calculated CF values for triply charged poly‐DL‐alanine cations at DF value of 250. Figure S4: Calibration graphs of CF values versus CCS from polynomial fit model of triply charged poly‐DL‐alanine cations using a DF values of 200 Td (blue), 220 Td (orange) and 250 Td (green). Figure S5: Full extracted CF scan for (a) the singly charged Bradykinin ion [M + H]+ (blue), where * is [M + H]+ solvent cluster [file RCM-39-e10010-s001.docx]

**Determination of collisional cross sections using microscale high-field asymmetric waveform ion mobility spectroscopy-mass spectrometry (FAIMS-MS).**

**ELECTRONIC SUPPLEMENTARY MATERIAL**

Kristina Krasnova,^a^ Colin S. Creaser, ^a^ and James C. Reynolds^a^*

^a^ Centre for Analytical Science, Department of Chemistry, Loughborough University, LE11 3TU, UK

* Corresponding author: [j.c.reynolds@lboro.ac.uk](mailto:j.c.reynolds@lboro.ac.uk)

**
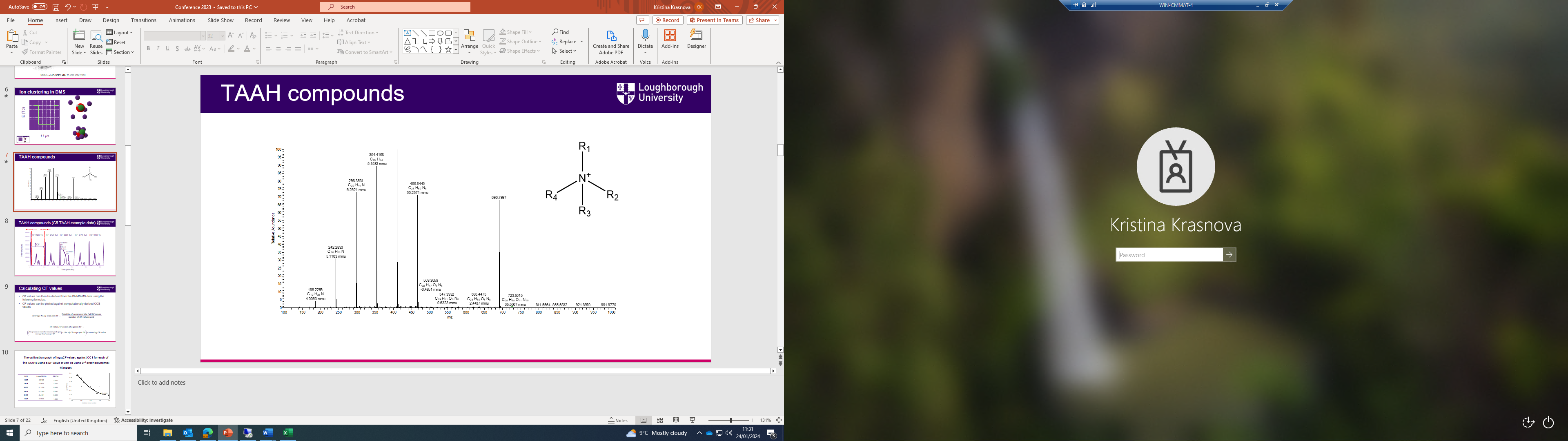
**

Figure S1: ESI-MS average mass spectra of TAAHs solutions at 1 µg/ml.

Table S1: Published CCS of tetraalkylammonium compounds (TAAHs) measured in nitrogen gas.^22^

| **TAAHs ions** | **Chemical Formula** | **Theoretical m/z** | **CCS Å^2^** |
| --- | --- | --- | --- |
| Tetrapropylammonium [M+] | C_12_H_28_N | 186.2222 | 145.7 |
| Tetrabutylammonium [M+] | C_16_H_36_N | 242.2848 | 167.5 |
| Tetrapentylammonium [M+] | C_20_H_44_N | 298.3474 | 182.7 |
| Tetrahexylammonium [M+] | C_24_H_52_N | 354.4100 | 213.1 |
| Tetraheptyammonium [M+] | C_28_H_60_N | 410.4726 | 233.9 |
| Tetraoctylammonium [M+] | C_32_H_68_N | 466.5352 | 254.8 |
| Tetradodecylammonium [M+] | C_48_H_100_N | 690.7856 | 316.9 |

Table S2: The percentage change in CCS of the Tetrahexylammonium halide ion between the published CCS value and those derived from a second order polynomial fit model using TAA^+^ calibrant ions with electrospray ionization.

| **DF (Td)** | **Mean CF (Td)** | **Predicted CCS (Å^2^)** | **Difference** | **% change** |
| --- | --- | --- | --- | --- |
| 200 | 0.8154 | 209.4 | -3.7 | -1.74 |
| 210 | 0.9123 | 206.3 | -6.8 | -3.19 |
| 220 | 0.9380 | 211.9 | -1.2 | -0.56 |
| 230 | 1.0615 | 206.7 | -6.4 | -3.00 |
| 240 | 1.3076 | 198.8 | -14.3 | -6.71 |
| 250 | 1.3076 | 205.1 | -8.0 | -3.75 |
| 260 | 1.4308 | 208.8 | -4.3 | -2.02 |
| 270 | 2.1692 | 187.0 | -26.1 | -12.25 |
| 280 | 2.2920 | 180.4 | -32.7 | -15.34 |

Table S3: The percentage change in CCS of the (Gly_5_) [M+H]^+^ ion between the published CCS value and those derived from a second order polynomial fit model using TAA^+^ calibrant ions with electrospray ionization.

| **DF (Td)** | **Mean CF (Td)** | **Predicted CCS (Å^2^)** | **Difference** | **% change** |
| --- | --- | --- | --- | --- |
| 200 | 0.7400 | 213.3 | 51.9 | 32.16 |
| 210 | 0.8700 | 208.3 | 46.9 | 29.06 |
| 220 | 1.0900 | 204.9 | 43.5 | 26.95 |
| 230 | 1.3100 | 196.5 | 35.1 | 21.75 |
| 240 | 1.3900 | 196.0 | 34.6 | 21.44 |
| 250 | 1.5800 | 195.3 | 33.9 | 21.00 |
| 260 | 1.9400 | 189.3 | 27.9 | 17.29 |
| 270 | 1.9700 | 194.5 | 33.1 | 20.51 |
| 280 | 2.4100 | 174.1 | 12.7 | 7.87 |

Figure S2: Calibration graph of transmission CF values vs CCS for TAA^+^ calibrant ions obtained at a DF value of 260 Td and used for CCS determination of the C6 TAA+ and (Gly)_5_ ions.

Table S4: The percentage change in CCS of the Tetrahexylammonium halide ion between the published CCS value and those derived from a second order polynomial fit model using TAA^+^ calibrant ions with nano-electrospray ionization.

| **DF (Td)** | **Mean CF (Td)** | **Predicted CCS (Å^2^)** | **Difference** | **% change** |
| --- | --- | --- | --- | --- |
| 200 | 0.8154 | 222.5 | 9.4 | **4.4144** |
| 210 | 0.9123 | 239.3 | 26.2 | **12.2933** |
| 220 | 0.9380 | 219.1 | 6.0 | **2.7987** |
| 230 | 1.0615 | 213.3 | 0.2 | **0.0788** |
| 240 | 1.3076 | 216.8 | 3.7 | **1.7518** |
| 250 | 1.3076 | 211.9 | -1.2 | **-0.5791** |
| 260 | 1.4308 | 213.2 | 0.1 | **0.0235** |
| 270 | 2.1692 | 204.0 | -9.1 | **-4.2633** |
| 280 | 2.2920 | 213.5 | 0.4 | **0.1779** |

Table S5: Published CCS of singly charged Poly-DL-Alanine cations measured in nitrogen gas and their *m/z*.^22^

| **Poly-DL-Alanine Peptides**  **(+1 charge state)** | **Theoretical m/z** | ***CCS* Å^2^** |
| --- | --- | --- |
|  |  |  |
| (Alanine)_6_ | 445.2410 | 194.0 |
| (Alanine)_7_ | 516.2782 | 209.7 |
| (Alanine)_8_ | 587.3153 | 226.2 |
| (Alanine)_9_ | 658.3524 | 239.9 |
| (Alanine)_10_ | 729.3895 | 252.5 |
| (Alanine)_11_ | 800.4266 | 265.7 |
| (Alanine)_12_ | 871.4637 | 278.5 |
| (Alanine)_13_ | 942.5008 | 290.8 |
| (Alanine)_14_ | 1013.5380 | 302.0 |
| (Alanine)_15_ | 1084.5751 | 313.7 |
| (Alanine)_16_ | 1155.6122 | 324.3 |
| (Alanine)_17_ | 1226.6493 | 335.8 |
| (Alanine)_18_ | 1297.6864 | 346.3 |
| (Alanine)_19_ | 1368.7235 | 358.4 |
| (Alanine)_20_ | 1439.7606 | 366.1 |
| (Alanine)_21_ | 1510.7978 | 376.5 |
| (Alanine)_22_ | 1581.8349 | 387.0 |
| (Alanine)_23_ | 1652.8720 | 393.0 |

Figure S3: Overlaid calibration data from ESI-FAIMS-MS and nano-ESI FAIMS-MS analysis of poly-DL-alanine obtained at a DF value of 280 Td.

Table S6: Published CCS of doubly charged Poly-DL-Alanine cations measured in nitrogen gas and their m/z.^22^

| **Poly-DL-Alanine  (+2 charge state)** | **Theoretical m/z** | **CCS Å^2^** |
| --- | --- | --- |
| (Alanine)_16_ | 578.3100 | 356.5 |
| (Alanine)_17_ | 613.8286 | 368.3 |
| (Alanine)_18_ | 649.3472 | 380.6 |
| (Alanine)_19_ | 684.8657 | 391.9 |
| (Alanine)_20_ | 720.3843 | 403.7 |
| (Alanine)_21_ | 755.9028 | 414.8 |
| (Alanine)_22_ | 791.4214 | 425.3 |
| (Alanine)_23_ | 826.9399 | 436.5 |
| (Alanine)_24_ | 862.4585 | 447.7 |
| (Alanine)_25_ | 897.9771 | 458.5 |
| (Alanine)_26_ | 933.4956 | 470.9 |
| (Alanine)_27_ | 969.0142 | 483.5 |
| (Alanine)_28_ | 1004.5327 | 494.6 |
| (Alanine)_29_ | 1040.0513 | 505.9 |

Table S7: Published CCS of triply charged Poly-DL-Alanine cations measured in nitrogen gas and their *m/z*.^22^

| **Poly-DL-Alanine**  **(+3 charge state)** | **Theoretical m/z** | **CCS Å^2^** |
| --- | --- | --- |
| (Alanine)_26_ | 622.6663 | 578.2 |
| (Alanine)_27_ | 646.3454 | 592.7 |
| (Alanine)_28_ | 670.0244 | 607.4 |
| (Alanine)_30_ | 717.3825 | 635.9 |
| (Alanine)_31_ | 741.0615 | 649.3 |

Table S8: Calculated transmission CF values for doubly charged Poly-DL-Alanine cations at DF value of 250 Td.

| **Poly-DL-Alanine** | ***CCS*** | **Mean CF (Td)** |
| --- | --- | --- |
| (Alanine)_16_ | 356.5 | 2.3901 |
| (Alanine)_17_ | 368.3 | 2.2482 |
| (Alanine)_18_ | 380.6 | 2.1064 |
| (Alanine)_19_ | 391.9 | 2.0118 |
| (Alanine)_20_ | 403.7 | 1.8700 |
| (Alanine)_21_ | 414.8 | 1.7754 |
| (Alanine)_22_ | 425.3 | 1.6809 |
| (Alanine)_23_ | 436.5 | 1.6309 |
| (Alanine)_24_ | 447.7 | 1.4863 |
| (Alanine)_25_ | 458.5 | 1.4418 |
| (Alanine)_26_ | 470.9 | 1.3972 |
| (Alanine)_27_ | 483.5 | 1.3026 |
| (Alanine)_28_ | 494.6 | 1.2553 |
| (Alanine)_29_ | 505.9 | 1.1608 |

Table S9: Calculated CF values for triply charged Poly-DL-Alanine cations at DF value of 250.

| **Poly-DL-Alanine** | **CCS Å^2^** | **Mean CF (Td)** |
| --- | --- | --- |
| (Alanine)_26_ | 578.2 | 2.3901 |
| (Alanine)_27_ | 592.7 | 2.2482 |
| (Alanine)_28_ | 607.4 | 2.1064 |
| (Alanine)_30_ | 635.9 | 1.9172 |
| (Alanine)_31_ | 649.3 | 1.8227 |

Figure S4: Calibration graphs of CF values vs CCS from polynomial fit model of triply charged Poly-DL-Alanine cations using a DF values of 200 Td (blue), 220 Td (orange), 250 Td (green).

**a)**

**b)**

230

270

260

240

250

*

260

250

240

230

220

210

200

○

*

Δ

210

200

270

280

220

280

Figure S5: Full extracted CF scan for a) the singly charged Bradykinin ion [M+H]^+^ (blue), where * is [M+H]^+^ solvent clusters and Δ is [M+H]^+^ monomer ions, and b) the doubly charged Bradykinin ion [M+2H]^2+^ (orange) over the range from -3 to 5 Td at 9 different DF values, where * is [M+2H]^2+^ solvent clusters, Δ and ○ are [M+2H]^2+^ monomer ions, which are conformational isomers of each other.

Table S10: The percentage change in CCS of the singly charged Substance P [M+H]^+^ between the published CCS value and those derived from polynomial fit model using Poly-DL-Alanine.

| **DF (Td)** | **Mean CF (Td)** | **Predicted CCS (Å^2^)** | **Difference (Å^2^)** | **% change** |
| --- | --- | --- | --- | --- |
| 200 | 0.3333 | 312.7980 | 49.2020 | **-13.5917** |
| 210 | 0.3333 | 313.8130 | 48.1870 | **-13.3113** |
| 220 | 0.4391 | 312.2180 | 49.7820 | **-13.7519** |
| 230 | 0.4921 | 319.0270 | 42.9730 | **-11.8710** |
| 240 | 0.6508 | 328.9660 | 33.0340 | **-9.1254** |
| 250 | 0.7037 | 343.8770 | 18.1230 | **-5.0064** |
| 260 | 0.8095 | 348.4950 | 13.5050 | **-3.7307** |
| 270 | 0.9154 | 339.3970 | 22.6030 | **-6.2439** |
| 280 | 0.9683 | 369.1270 | -7.1270 | **1.9688** |

Table S11: The percentage change in CCS of the doubly charged Substance P ion [M+2H]^2+^ between the published CCS value and those derived from polynomial fit model using Poly-DL-Alanine.

| **DF (Td)** | **Mean CF (Td)** | **Predicted CCS (Å^2^)** | **Difference (Å^2^)** | **% change** |
| --- | --- | --- | --- | --- |
| 200 | 0.9683 | 386.7750 | 13.2250 | **-3.3063** |
| 210 | 1.1270 | 377.7950 | 22.2050 | **-5.5513** |
| 220 | 1.2857 | 386.2780 | 13.7220 | **-3.4305** |
| 230 | 1.4444 | 409.1670 | -9.1670 | **2.2917** |
| 240 | 1.6032 | 406.4140 | -6.4140 | **1.6035** |
| 250 | 1.7619 | 409.8330 | -9.8330 | **2.4583** |
| 260 | 1.9206 | 418.8190 | -18.8190 | **4.7048** |
| 270 | 2.2381 | 422.7590 | -22.7590 | **5.6898** |
| 280 | 2.3968 | 420.0050 | -20.0050 | **5.0013** |

**b)**

**a)**

280

270

0

260

250

240

240

Δ

*

Δ

230

220

210

200

280

270

260

250

*

220

230

210

200

Figure S6: Full extracted CF scan for a) the singly charged Substance P ion [M+H]^+^ (blue), where * is [M+H]^+^ solvent clusters and Δ is [M+H]^+^ monomer ions, and b)

the doubly charged Substance P ion [M+2H]^2+^ (orange) over the range from -3 to 5 Td at 9 different DF values, where * is [M+2H]^2+^ solvent clusters, Δ is [M+2H]^2+^ monomer ions.
